# Giant Fibroepithelial Polyp of the Penis

**DOI:** 10.7759/cureus.50204

**Published:** 2023-12-08

**Authors:** Boris Kangabam, Amitkumar Khwairakpam, Yengkhom Daniel Singh, Babina Sorokhaibam

**Affiliations:** 1 Urology, Regional Institute of Medical Sciences, Imphal, IND; 2 Pathology, Regional Institute of Medical Sciences, Imphal, IND

**Keywords:** bladder cancer, radiation, scrotal edema, penis and scrotum, penis, prepuce, fibroepithelial polyp

## Abstract

A fibroepithelial polyp (FEP) of the penis is a rare benign swelling and often under-recognised lesion that has the potential to become malignant in some cases. The pathogenesis is still unclear, but it is hypothesised to be due to chronic irritation most often associated with condom catheter use or phimosis. We describe a case of an FEP measuring 10 cm in largest diameter developing from the ventral prepuce with a longstanding post-radiation penoscrotal oedema. A 62-year-old male with a history of bladder cancer presented to the emergency department with abdominal distention, vomiting, and obstipation for three days to the emergency department. He had post-radiation penoscrotal edema for the last 10 years and penile tip growth for the last two years. Foley's catheter insertion was done through the urethra after dorsal slit of prepuce, and an incisional biopsy was sent, which was found to be an FEP.

## Introduction

A fibroepithelial polyp (FEP) of the penis was initially documented by J.F. Fetsch et al. in 2004 [1]. This condition is frequently underestimated and infrequently observed originating from the penis. FEPs are non-malignant mesodermal tumors covered with the epithelium, characterized by a fibrovascular stroma and emerging from the submucosa [2]. To date, fewer than 30 cases have been documented in the English literature. The primary cause is generally associated with condom catheter use. Uncommon triggers encompass paraphimosis, trauma such as genital hanging kung fu, congenital anomalies, prolonged pad use, and, in some instances, idiopathic [3]. Here, we present a unique case involving a sizeable FEP on the prepuce of a 62-year-old male with a history of Signet ring cell carcinoma of the bladder, experiencing penoscrotal edema post-adjuvant radiotherapy, not associated with condom catheter use and paraphimosis. Notably, this particular etiology has not been previously reported as a causative factor for FEPs in the English medical literature.

## Case presentation

**Figure 1:**
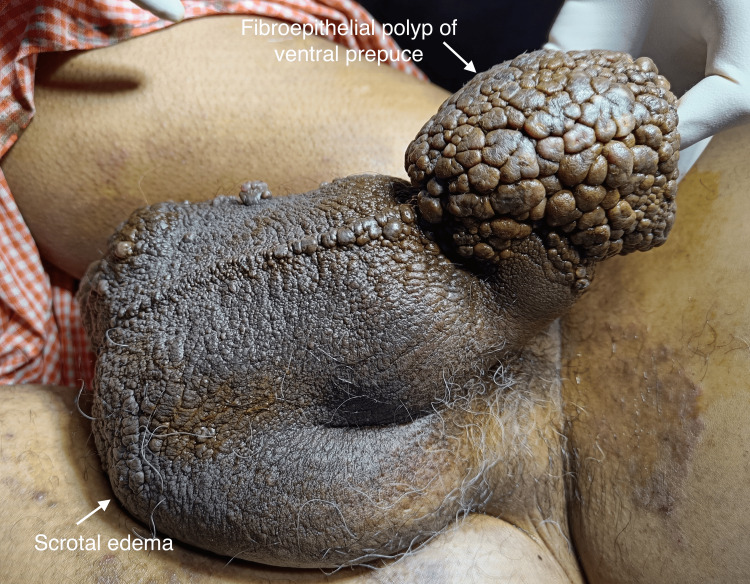
Clinical picture of a giant polypoidal lesion measuring 10 cm in its maximum dimension and involving the ventral prepuce. Accompanying bilateral scrotal edema seen.

A 62-year-old male with a known case of signet ring cell bladder carcinoma presented with features of acute abdominal obstruction to the emergency department. He had a history of transurethral resection of bladder tumour (TURBT) 13 years ago, followed by adjuvant radiation therapy with a total dose of 58 Gy. He had been advised to do a radical cystectomy but he refused. He developed post-radiation penoscrotal oedema for the last 10 years and a slowly expanding growth at the penile tip for the last two years. He had post-micturition dribbling. Otherwise, there were no significant lower urinary tract symptoms, or history of trauma, diabetes mellitus, tuberculosis, hypertension or recent travel, which may predispose him to filariasis. There was no history of use of condom catheters in the past. On examination, the abdomen was distended, and bilateral lower limbs non-pitting oedema and penoscrotal oedema were present. There was a cauliflower-like, firm, solid, non-tender growth over the ventral prepuce measuring around 10 cm × 5 cm × 2 cm, with multiple small grape-like lesions over the penile shaft (Figure 1).

**Figure 2:**
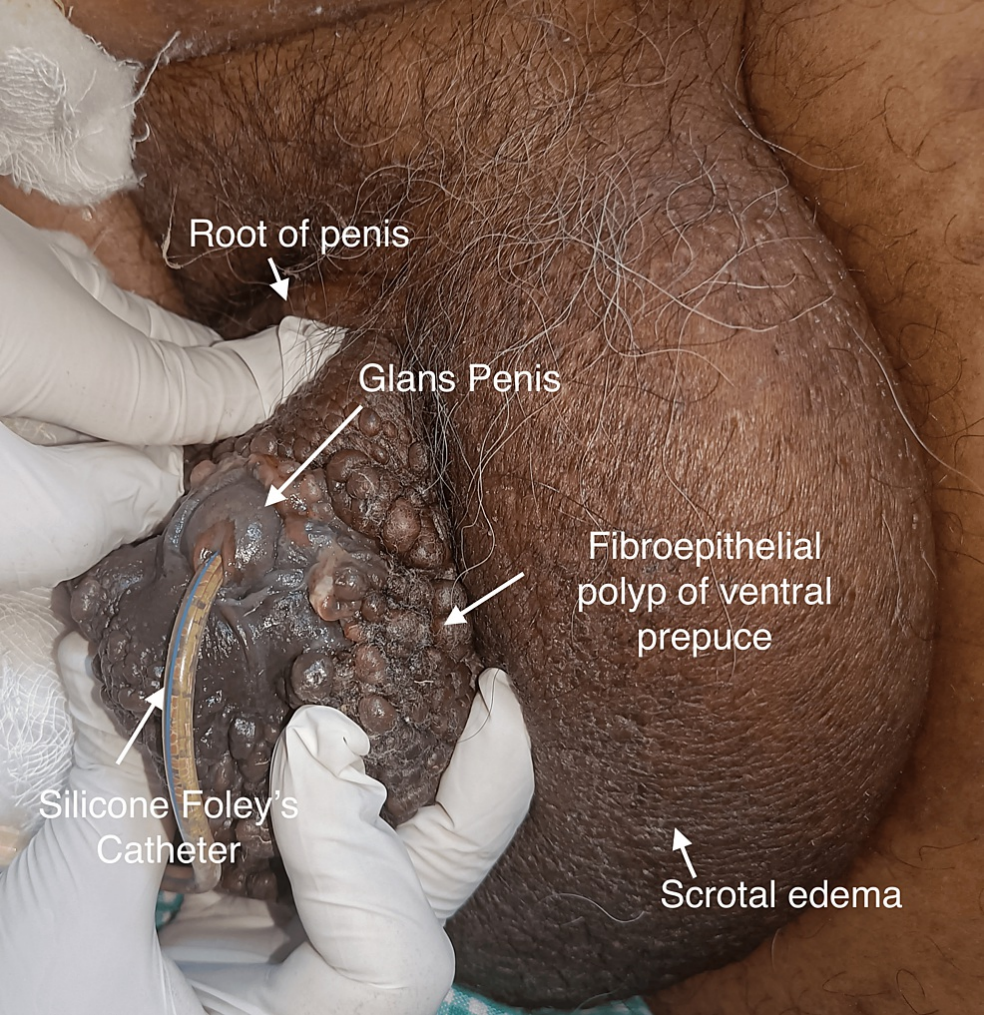
Glans penis seen after the dorsal slit of the prepuce with Foley’s urethral catheter in situ

**Figure 3:**
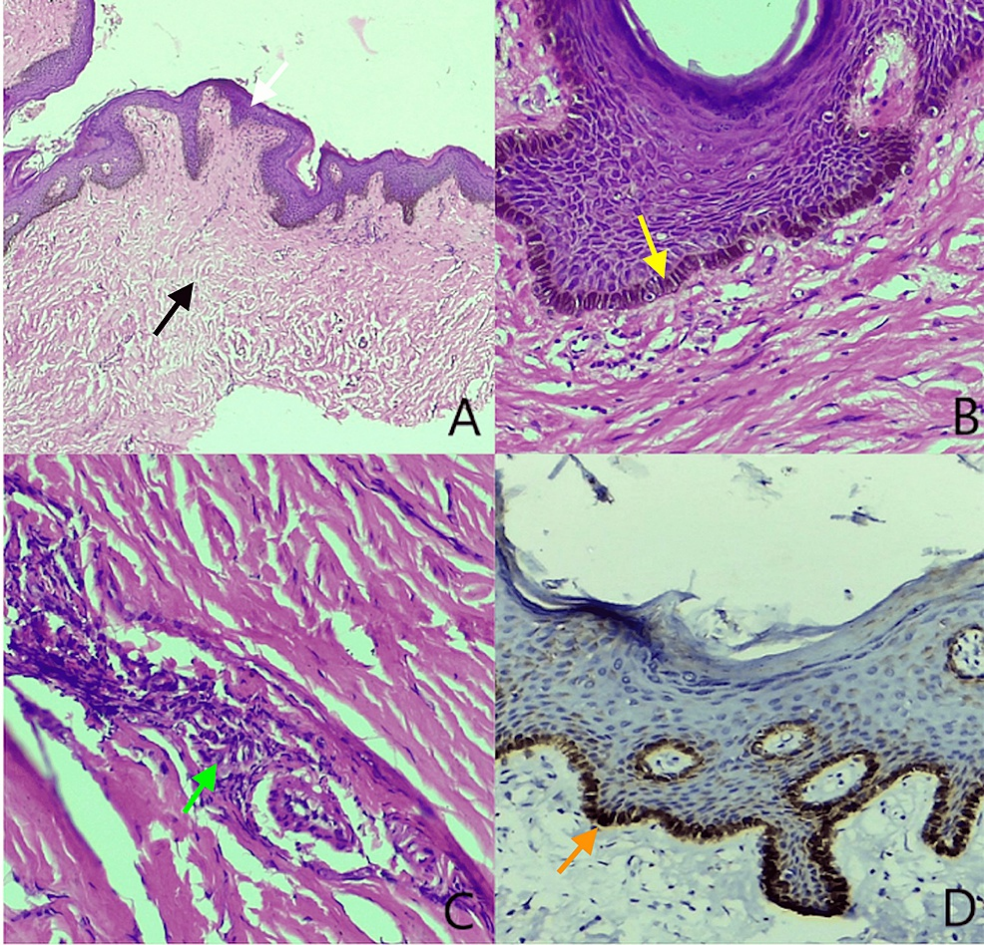
Histopathological images of the giant fibroepithelial polyp (clockwise A-D): A) Hyperkeratotic, acanthotic epidermis (white arrow) overlying loose fibrocollagenous stroma (black arrow) (hemotoxylin and eosin (H&E), 40x). B) Increased basal melanin (yellow arrow) (H&E, 200x). C) Focal sparse perivascular lymphocytic infiltrate(green arrow) (H&E, 200x). D) p16 immunohistochemistry showing increased cytoplasmic and nuclear staining (orange arrow).

External urethral meatus was not visible as the prepuce could not be retracted due to extensive growth. On examination after the dorsal slit, glans and urethra appeared to be spared from the lesion (Figure 2). Foley's urethral catheter was inserted after the dorsal slit. There was no palpable inguinal lymphadenopathy. Routine blood and urine tests were unremarkable. Peripheral blood smears were negative for filarial worms. He tested negative for human immunodeficiency virus. Ultrasonography of the inguinoscrotal region revealed a gross distended scrotum with wall thickening, suggestive of lymphedema likely to be post-radiation therapy changes. Preoperatively, an incisional biopsy from the dorsolateral part was taken near the edge of the swelling. Exploratory laparotomy and loop ileostomy were done. Intraoperatively, there were adhesions between the posterior wall of the rectum to the sacral promontory with hugely dilated small and large bowels. For the FEP management, the patient declined any surgical treatment. Histopathologic examination of the preputial incisional biopsy showed a polypoidal skin lesion with a hyperkeratotic, mildly acanthotic epidermis with increased basal melanocytes. The underlying loosely fibrocollagenous stroma showed many dilated blood vessels with sparse lymphocytes. No evidence of dysplasia was noted (Figure 3). The final interpretation was an FEP. Immunohistochemistry for p16 showed increased staining on the basal keratinocytes.

The patient had an uneventful hospital recovery and was discharged after 10 days. Ultrasonography of the abdomen done after 10 days postoperatively revealed no significant abnormality. Surgical site wounds were healthy with a functioning ileostomy. The patient was advised to review in the urology outpatient department for definitive treatment of the penile swelling. However, the patient did not turn up in subsequent visits and was lost to follow-up.

## Discussion

FEPs arise rarely from the penis. It is known by a myriad of names, such as soft fibroma, fibroma molle, acrochordon, fibroma pendulans, or simply as a skin tag [4]. As to the urinary tract, they are encountered more in the ureter and renal pelvis and rarely in the posterior urethra or bladder [2]. FEPs of the penis differ from the usual cutaneous FEP in that they are larger, with notable stromal oedema and vascular dilation and more stromal cellularity. Skin tags are usually small with a size of less than 5 mm commonly seen in the axilla, neck, groin, eyelid, and inframammary regions [4]. Median age and size at presentation for penile FEPs are 40 years and 2.5 cm, respectively, based on a series by Fetsch et al. [1].

The pathogenesis of the formation of FEPs is unclear. It may be due to chronic irritation that involves sub-epithelial stroma, rather than a true neoplasm [4]. Another possibility is based on the expression of hormone receptors by the stromal cells of FEPs, such as estrogen and progesterone receptors. Thus, hormonal influences could be contributing to the pathogenesis of these lesions [5]. However, in cases where hyperplasia and hormone receptors do not account for its formation, inflammation may contribute to the pathogenesis of lymphedematous FEP [5]. Patients with radiation will have lymph node fibrosis with mechanical insufficiency of vessels and decreased proliferative capability, thereby causing lymphedema of the region drained by the affected lymph node [6]. Persistent lymphedema promotes fibrosis and inflammation [6]. Hence, in our case with no other known risk factors for FEP, radiation-induced penile oedema was likely to be the triggering factor for the development of FEP. Notably, there have been reports of the presence of virus like human herpes virus (HHV)-8 detected by the HHV-8 sequence in the lesions via a polymerase chain reaction [2]. Immunostaining for HHV-8 was not positive as only a few cells were stained. However, its contribution to the formation of the lesion could not be excluded. The relation between HPV and FEP is controversial. Gupta et al. [7] found correlation between FEP and low-risk HPV types 6 and 11. By contrast, Pezeshkpoor et al. [8] concluded that there was no relationship between FEP and HPV. In our case, as p16 was positive on immunohistochemistry, which might suggest a possible correlation with HPV and needs further studies.

Although described as benign, FEPs of the penis require treatment as they may undergo malignant transformation into squamous cell carcinoma as reported in some cases [9]. Moreover, the mass may become a nidus for infection if the tissue mass becomes devitalised. Conservative local excision under local anaesthesia is the usual form of treatment, but it may recur if the inciting factor persists [4].

## Conclusions

An FEP of the prepuce is a rare benign swelling and should be considered in the differential diagnosis of penile neoplasms. We present a case of an FEP of the prepuce due to its rarity and unusual cause. It may occur in a setting of long-standing post-radiation induced penile edema. A thorough history of the patient should be taken to identify the cause and to reduce chance of recurrence in the future by removing the inciting factor. Diagnosis requires inspection and suspicion, and all such swellings should be biopsied and sent for histopathology. There might be a chance of conversion to malignancy. Therefore, treatment is recommended for any such swellings. The usual treatment of the FEP of the penis is excision of the swelling under local anaesthesia.
